# A comparative analysis of risk factor associations with interval and screen-detected breast cancers: A large UK prospective study

**DOI:** 10.1002/ijc.34968

**Published:** 2024-04-26

**Authors:** Isobel Barnes, Montserrat Garcia-Closas, Toral Gathani, Siân Sweetland, Sarah Floud, Gillian K. Reeves

**Affiliations:** 1 Cancer Epidemiology Unit, Nuffield Department of Population Health, University of Oxford, Oxford, UK; 2 Division of Genetics and Epidemiology, Institute of Cancer Research, London, UK; 3 Oxford University Hospitals NHS Foundation Trust, Oxford, UK

**Keywords:** breast cancer, interval cancer, screen-detected cancer, screening

## Abstract

The associations of certain factors, such as age and menopausal hormone therapy, with breast cancer risk are known to differ for interval and screen-detected cancers. However, the extent to which associations of other established breast cancer risk factors differ by mode of detection is unclear. We investigated associations of a wide range of risk factors using data from a large UK cohort with linkage to the National Health Service Breast Screening Programme, cancer registration, and other health records. We used Cox regression to estimate adjusted relative risks (RRs) and 95% confidence intervals (CIs) for associations between risk factors and breast cancer risk. A total of 9421 screen-detected and 5166 interval cancers were diagnosed in 517,555 women who were followed for an average of 9.72 years. We observed the following differences in risk factor associations by mode of detection: greater body mass index (BMI) was associated with a smaller increased risk of interval (RR per 5 unit increase 1.07, 95% CI 1.03–1.11) than screen-detected cancer (RR 1.27, 1.23–1.30); having a first-degree family history was associated with a greater increased risk of interval (RR 1.81, 1.68–1.95) than screen-detected cancer (RR 1.52, 1.43–1.61); and having had previous breast surgery was associated with a greater increased risk of interval (RR 1.85, 1.72–1.99) than screen-detected cancer (RR 1.34, 1.26–1.42). As these differences in associations were relatively unchanged after adjustment for tumour grade, and are in line with the effects of these factors on mammographic density, they are likely to reflect the effects of these risk factors on screening sensitivity.

## 1 Introduction

In the United Kingdom, the National Health Service (NHS) Breast Screening Programme offers routine mammographic screening every 3 years to all women aged 50–70. Among women who attend for routine mammography around two thirds of breast cancers are detected at screening, and around one third are detected symptomatically in the interval between screens.^1^

An interval cancer may arise because it was missed at screening. Alternatively, it may arise because it was an aggressive tumour that was too small to be detected at screening, but quickly became symptomatic. As such, differences in associations of a given risk factor with interval versus screen-detected breast cancers are an indication that the risk factor may impact on the ability of screening to detect cancers (i.e., screening sensitivity) and/or that it has a differential effect on more or less aggressive tumours. This information can be used to identify women who are at greatest risk of interval cancers, and who may therefore benefit from alternative screening interventions. It can also be used to help understand differences in the aetiology of interval and screen-detected cancers.

Certain risk factors for breast cancer, including age and use of menopausal hormone therapy (MHT), have been consistently shown to have different associations with interval and screen-detected breast cancers. Increasing age is associated with a smaller increased risk of interval than of screen-detected cancer,^2–4^ and use of MHT is associated with a greater increased risk of interval cancers than of screen-detected cancers.^5–8^ These differences are likely to be explained by the effects of these factors on breast density. Breast density is a measure of the amount of fibroglandular tissue in the breast and since fibroglandular tissue is radiographically opaque, it is harder to detect solid tumours using mammography in women with dense breasts.^9^ Since decreasing age and use of MHT are both associated with greater breast density,^10,11^ they are thought to be associated with a greater risk of interval than screen-detected cancers because they decrease screening sensitivity.^5,7,12^

The comparative associations of other risk factors for breast cancer with interval versus screen-detected cancers, are less clear. Four prospective studies,^5–7,13^ and a larger number of studies which reported associations from case–control and case-only analyses, have presented relevant findings for body mass index (BMI), reproductive factors, alcohol intake, and family history of breast cancer^8,14–19^ but their findings are inconsistent, possibly due to differences in the populations studied in terms of MHT use as its effect on breast density may mask the comparatively lesser effects of other risk factors. Further large-scale prospective evidence based on women who have never used MHT is therefore needed.

We present findings from an analysis of over half a million post-menopausal never MHT users in the Million Women Study, a large ongoing UK cohort study, which aims to describe the characteristics of interval and screen-detected cancers, and to compare associations of established breast cancer risk factors with interval versus screen-detected breast cancers.

## 2 Materials and Methods

### 2.1 The Million Women Study

Between 1996 and 2001, around 1.3 million women invited for NHS breast cancer screening at 66 screening centres in England and Scotland, were recruited to the Million Women Study. Participants received a questionnaire alongside their letter of invitation to routine mammography. The questionnaire was returned when the woman came for screening and subsequent questionnaires were sent at ~3–5 yearly intervals. The design, methods, questionnaires, and data access policies for the Million Women Study are available at www.millionwomenstudy.org.

All study participants are followed for deaths, cancer registrations, and emigration through linkage to data routinely collected by the NHS. Data are provided through NHS England and Public Health Scotland. The linked cancer registration records include cancer type and site coded to the 10th Revision of the International Classification of Diseases (ICD-10), with histology coded to ICD-O-3 or 4. For women with a registration of breast cancer, the cancer registration records included information on mode of detection (screen-detected or interval). Information on tumour characteristics was derived from cancer registration records and, in some cases, using additional information from medical records and questionnaire data.

### 2.2 The NHS Breast Screening Programme

In 1988, the NHS Breast Screening Programme (NHSBSP) began inviting all women aged 50–64 years and registered with a general practitioner for routine mammographic screening once every 3 years. Between 2001 and 2004, the NHSBSP implemented an increase in the upper age limit from 64 to 70.

The NHSBSP central databases hold individual information on women’s screening episodes. A screening episode is defined as the period of time during which all activities relating to screening, including invitation and diagnostic assessment, take place and the information held includes the date the episode started and whether or not a woman attended for screening. In 2013, the Million Women Study cohort was linked to breast cancer screening records for women in England by NHS Connecting for Health (now superseded by NHS England) using the NHS number and date of birth. This provided information on dates and attendance for all screening episodes in Million Women Study participants from the start of the NHSBSP to the end of 2012.

### 2.3 Breast cancer risk factors

All information on breast cancer risk factors came from the Million Women Study recruitment questionnaire. We considered the following factors: age at menarche (<12, 12–13, 14 years), parity (nulliparous, 1–2, 3+ full-term births), age at first birth (nulliparous, <25 years, 25+ years), BMI (<25, 25–29, 30+ kg/m^2^), alcohol (<3, 3–7, 8+ units/week), smoking (never, past, current), first-degree family history of breast cancer (no, yes), and previous surgery for benign breast disease (no, yes).

### 2.4 Outcomes

The two main outcomes of interest were interval and screen-detected invasive breast cancers (ICD10: C50). Information on mode of detection was only available for 62% of breast cancer registrations that occurred after a woman had attended for a screening episode. Since most screen-detected cancers are registered shortly after the start of a screening episode, we used the time from the start of the episode to cancer registration as a marker for interval versus screen-detected cancers. We defined interval cancers as those registered 9 months after an attended routine breast screen, but before the start of the next routine screening episode. Screen-detected cancers were defined as those registered within 9 months of an attended routine screening episode. We chose a cut-off of 9 months as it was associated with the point on the receiver operator characteristic curve closest to the left-hand corner of the plot correctly identifying 95% of interval cancers and 97% of screen-detected cancers (Figure S1). This point provides an optimal cut-off under the assumption that the cost of making a false-positive result is the same as the cost of making a false-negative result.^20^ In sensitivity analyses aimed at assessing the robustness of the findings to the choice of cut-off used to define whether a breast cancer was screen-detected or interval, we repeated the main analyses using a cut-off of 6 months instead of 9 months. This alternative cut-off was chosen on the basis that the NHSBSP aims for breast screening episodes to last no longer than 6 months.^21^

Breast cancers were additionally classified according to tumour size (<20, 20–49, 50+ mm), nodal involvement (negative, positive), TNM stage (1, 2, 3+), grade (1, 2, 3), histological subtype (ductal, lobular, other), and oestrogen receptor (ER) status (positive, negative).

### 2.5 Statistical analysis

Our analyses included only postmenopausal women, defined as women who had a natural menopause, a bilateral oophorectomy or were aged at least 55 years, since 96% of women with known age at menopause reported being postmenopausal at age 55.^22^ We excluded women who: reported ever using MHT on the Million Women Study recruitment questionnaire or on the subsequent 3- and 8-year questionnaires; had any previous registration for cancer (other than non-melanoma skin cancer, ICD-10 C44); and those that lived in Scotland at the time of recruitment as screening records were only available for women in England.

In order to characterise interval and screen-detected cancers in terms of their tumour characteristics, we first conducted logistic regression models among breast cancer cases only, in which the binary outcome was mode of detection. The results are presented in the form of odds ratios (ORs) and 95% confidence intervals (CIs) for interval versus screen-detected breast cancer according to categories of each tumour characteristic considered after adjustment for age at the start of the screening episode (<60, 60–64, 65+ years), region of recruitment (Oxford, East Anglia, South West, Thames, West Midlands, North Yorkshire, Trent, North West [Mersey], North West [Manchester/Lancashire]) and area deprivation quintile (based on the Townsend Index^23^).

We used Cox regression models to estimate risk factor associations separately within each type of risk period (interval and screen-detected). Women only contributed person-years to the analysis during the period for which we had linked information on screening episodes (i.e., up to 31 December 2012), and during screening episodes where they had attended screening. Women were followed from the date of recruitment if they reported having had a natural menopause or a bilateral oophorectomy at recruitment or were aged 55 years or older at recruitment. Otherwise, women were followed from their 55th birthday. Follow-up ended on the date of first cancer registration, date of death, date of cessation of NHS registration, or 3 years after the start of the last routine screening episode on record, whichever was earliest. We divided follow-up time according to whether women were at risk of an interval cancer or at risk of a screen-detected cancer-based on their screening records (Figure S2). Following our definition of interval and screen-detected cancers, the intervals between the start of successive screening episodes were divided using a cut-off of 9 months to distinguish between periods when women were at risk of an interval cancer and when they were at risk of a screen-detected cancer. Women who did not attend for a routine screen, exited analyses at the start of the missed/non-attended screening episode and re-entered at the start of the next attended screening episode. Results are presented as hazard ratios (hereafter referred to as relative risks [RRs]) and 95% CIs. Analyses were stratified by year of birth (≤1930, 1931, …, 1950+) and year of recruitment (1996, 1997, …, 2001+) and adjusted for cancer registry region, area deprivation quintile and, where appropriate, for all other risk factors.

In order to quantify relative differences in risk factor associations by mode of detection, we also conducted case-only logistic regression models of the risk of interval versus screen-detected breast cancer in relation to each risk factor, with adjustment for age at the start of the screening episode, cancer registry region, area deprivation quintile and, where appropriate, for all other risk factors. We assessed the extent to which any apparent association of a given risk factor with mode of detection was likely to be due to its association with screening sensitivity rather than tumour aggressiveness, by repeating these analyses with additional adjustment for grade which is an intrinsic marker of tumour aggressiveness.

To examine the impact on the main findings of the choice of cut-off used to define whether a cancer was interval or screen-detected, we also repeated our main analysis using a 6 month rather than a 9 month cut-off.

Missing values for adjustment variables were included as a separate category. Trends in RRs or ORs were calculated for ordinal risk factors (age at menarche, parity, BMI, and alcohol consumption) and tumour characteristics (tumour size, stage, and grade). This was done using the median value within each category of the risk factor or tumour characteristic. Competing risk methods were used to assess heterogeneity in RRs by mode of detection.^24^ Analyses were conducted using the statistical package Stata, version 17.0.^25^

## 3 Results

A total of 517,555 postmenopausal women in England without prior cancer (other than non-melanoma skin cancer) and who did not report using MHT were followed for invasive breast cancer. Table 1 presents their characteristics at baseline and details of their follow-up. During an average follow-up of 9.72 years per woman, 9421 screen-detected and 5166 interval breast cancers occurred.

Estimated ORs for interval cancers versus screen-detected cancers in relation to tumour characteristics are given in Table 2. Data on histological subtype were available for all cancers but the data on other tumour characteristics were relatively incomplete with the percent missing ranging from 8% for grade to 64% for ER status. There were significant differences between interval and screen-detected cancers for all of the tumour characteristics considered. Interval cancers were more likely than screen-detected cancers to be: larger (OR per 1 mm increase in tumour size 1.05, 95 %CI 1.05–1.06); node positive (OR 2.32, 2.11–2.54); of advanced stage (OR per unit increase in stage 2.25, 2.10–2.42); of higher grade (OR per unit increase in tumour grade 2.15, 2.03–2.27); lobular subtype (OR for lobular versus ductal 1.23, 1.10–1.37); and ER negative (OR 2.27, 1.95–2.64).

**Figure 1:**
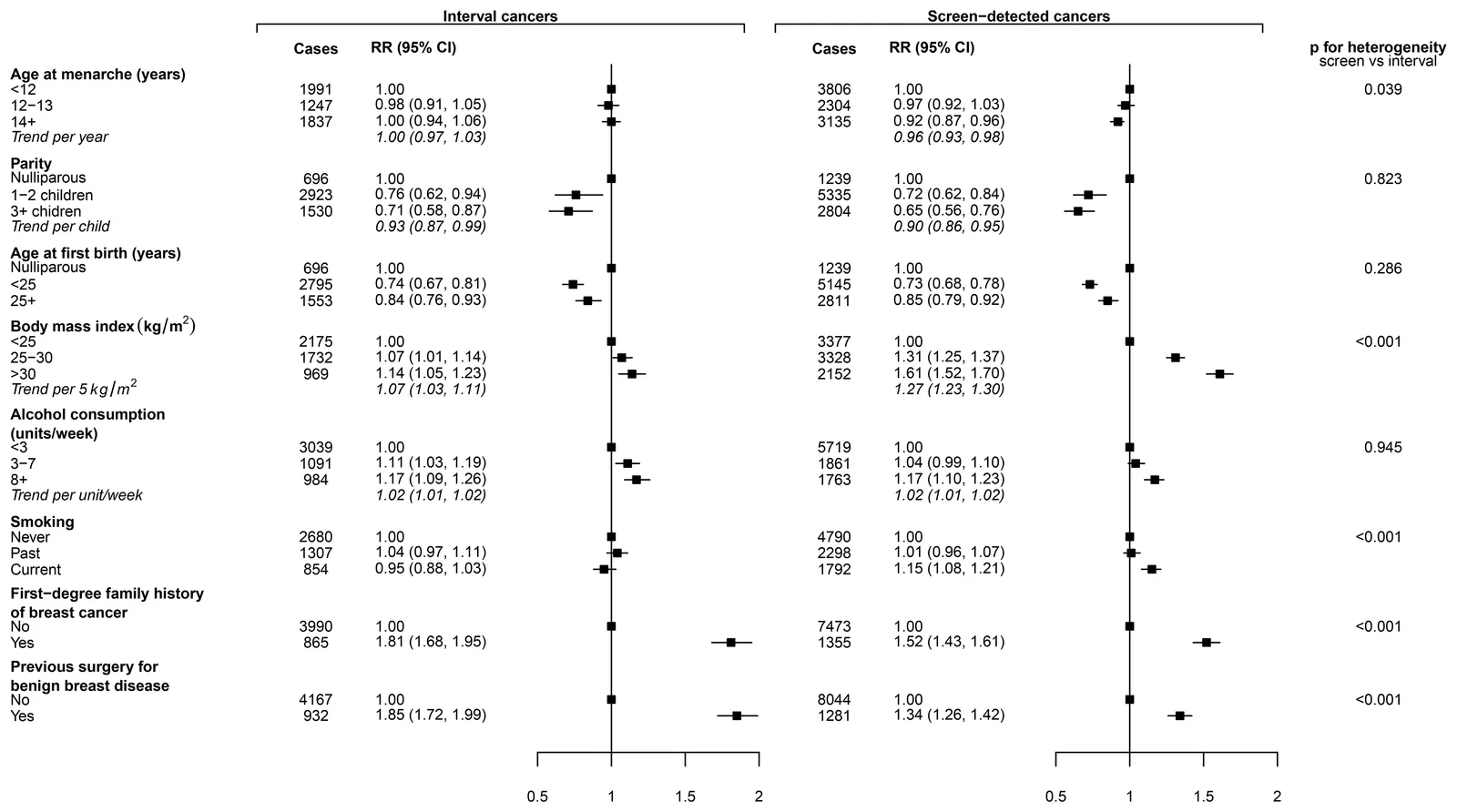
Relative risks (95% CIs) for interval breast cancer and screen-detected breast cancer in relation to established risk factors. Relative risks stratified by year of birth, year of recruitment; adjusted for region, deprivation quintile and risk factors listed above. P-values for heterogeneity are based on trends for age at menarche, parity, body mass index, and alcohol consumption. RR, relative risk; CI, confidence interval.

Figure 1 presents the RRs for interval cancer and screen-detected cancer in relation to established risk factors for breast cancer. There was strong evidence that the associations with risk of interval cancer differed to those with risk of screen-detected cancer for BMI, history of breast cancer in a first-degree relative, previous breast surgery, and smoking (all *p* for heterogeneity <.001). Although increasing BMI was associated with an increased risk of both interval and screen-detected cancer, the magnitude of the association was smaller for interval cancer (RR per 5 unit increase in BMI 1.07, 95% CI 1.03–1.11) than for screen-detected cancer (RR 1.27, 1.23–1.30). Current smoking was not associated with interval cancers (RR for current versus never smoking 0.95, 0.88–1.03), but was associated with an increased risk of screen-detected cancer (RR 1.15, 1.08–1.21). The increased risk of interval cancer associated with having a first-degree family history of breast cancer (RR 1.81, 1.68–1.95) was greater than for screen-detected cancer (RR 1.52, 1.43–1.61). Similarly, the increased risk associated with having had previous surgery for benign breast disease was greater for an interval cancer (RR 1.85, 1.72–1.99) than for screen-detected cancer (RR 1.34, 1.26–1.42).

Table 3 presents the ORs obtained from the case-only logistic regression analysis of each risk factor in relation to mode of breast cancer detection. A 5-unit increase in BMI, and being a current smoker as opposed to a never smoker, were associated with a 15% (95% CI 11%–19%) and 20% (12%–28%) lower risk of having an interval versus a screen-detected cancer, respectively. In contrast, a first-degree family history of breast cancer, and a history of benign breast disease, were associated with increases of 20% (9%–32%) and 38% (25%–52%), respectively, in the risk of having an interval versus a screen-detected cancer. Further adjustment for tumour grade had little impact on any of the estimated ORs.

There was no material difference between the results from analyses using a 9 month cut-off and analyses using a 6month cut-off (Table S1 and Figure S3).

## 4 Discussion

Using data from a large prospective UK cohort with 9421 screen-detected and 5166 interval breast cancers, we have shown that interval breast cancers were substantially more likely than screen-detected cancers to be larger, node positive, of advanced stage, higher grade, lobular subtype, and ER negative. Our finding that interval cancers have a generally more aggressive tumour characteristic profile than screen-detected cancers is in agreement with several previous studies.^15–17,26^

In our examination of risk factors, we found that the association between increasing BMI and increasing risk of breast cancer was smaller for interval cancers than for screen-detected cancers; current smoking was not associated with the risk of interval cancer, but was associated with an increased risk of screen-detected cancer; and both a first-degree family history of breast cancer and previous surgery for benign breast disease had greater positive associations with interval cancers than with screen-detected cancers. These differences in risk factor associations by mode of detection were relatively unchanged after adjustment for tumour grade.

The only two previous prospective studies to have assessed the association of BMI separately with interval and screen-detected breast cancer risk were the Cancer Prevention Study (CPS) II^5^ and a case–control study nested within the Melbourne Collaborative Cohort Study (MCCS).^13^ The findings of the CPS II study, which included 1281 interval and 2711 screen-detected cancers, are consistent with ours in that they reported a significantly smaller BMI-associated risk for interval cancer (RR per 5 unit increase 0.89, 95% CI 0.79–0.99) than for screen-detected cancer (1.06, 0.99–1.13; *p*-value for heterogeneity by mode of detection = 0.01). However, in contrast to our findings, they reported a marginally significant inverse association with interval cancer. The MCCS nested case–control study included 148 interval cancers and 244 screen-detected cancers and allowed associations of BMI with risk to vary by age by modelling an interaction between BMI and age. They reported an almost null association of BMI with both interval cancer (OR per 1 standard deviation increase 0.92, 95% CI 0.63–1.35) and screen-detected cancer (0.92, 0.66–1.27) at age 50. More in keeping with our results, they reported a smaller, but non-significant, positive association of BMI with interval cancer (1.12, 0.84–1.49) than with screen-detected cancer (1.29, 1.07–1.56) at age 70. Our finding of a significant positive association of BMI with the risk of both interval and screen-detected breast cancer reflects the established causal association of increased BMI with postmenopausal breast cancer incidence.^27^ Furthermore, the 28% lesser association of obesity with interval versus screen-detected cancers observed here is likely to reflect the substantial inverse association of BMI with breast density, in that women with higher BMI have lower breast density and so are more likely to have their cancer detected on a mammographic x-ray.^28^

To our knowledge, the CPS II study was also the only previous prospective study to have reported on the associations of smoking with interval and screen-detected cancer. They found that current smoking, compared to never smoking, was associated with an increased risk of interval cancers (RR 1.39, 95% CI 1.09–1.78) but not with screen-detected cancers (RR 1.10, 0.90–1.34), with no significant difference in associations by mode of detection. These findings are in contrast to those presented here, which suggest no association of smoking with interval cancers, but a modest positive association with screen-detected cancers, and a 20% lesser association of current smoking with interval versus screen-detected cancers. Our findings are in keeping with some previous evidence which suggests that smoking may reduce breast density,^29–31^ and merit further investigation to evaluate whether the current smoking association with breast cancer is, at least in part, mediated by its effect on mammographic screening sensitivity.

Three prospective studies, including the CPS II study and two large cohort studies based on women attending national breast screening programmes in Spain^6^ and Norway,^7^ have reported on family history in relation to interval and screen-detected breast cancer. The Spanish study included 1653 interval and 5309 screen-detected cancers, and the Norwegian study included 814 interval and 1512 screen-detected cancers. None of the three studies reported a significant difference in the associations of family history by mode of detection. However, the relative risks reported in the Norwegian breast screening study for interval (RR 1.72, 95% CI 1.23–2.40) and screen-detected cancers (RR 1.52, 1.17–1.96) were similar in magnitude to our estimates. Our finding of a 20% greater risk of interval versus screen-detected cancers in women with a family history of breast cancer could plausibly reflect either greater breast density in such women,^32–35^ or a greater association of family history with more aggressive disease. However, given that this finding was relatively unaffected by adjustment for tumour grade, and that previous studies have reported greater breast density in those with a positive family history, the more likely explanation is that women with a family history are less likely to have an existing tumour detected at mammographic screening. It is also possible that women with a family history of breast cancer are more breast aware and thus more likely to present symptomatically in the interval between screens. However, little empirical evidence is available about this conjecture.

The only study to have reported on markers of benign breast disease in relation to interval and screen-detected breast cancer was the Spanish breast screening study^6^ which found no significant difference in the associations of a woman’s previous history of breast biopsy by mode of detection, although the magnitude of the RRs for interval cancers (RR 1.73, 95% CI 1.46–2.04) and screen-detected cancers (RR 1.34, 1.18–1.53) were similar to those reported here. Previous surgery for benign breast disease is not known to affect breast density but our finding of a 38% increase in risk of interval versus screen-detected cancers in women with previous treated benign breast disease is possibly due to post-operative mammographic changes which could reduce screening sensitivity.^36^ As with a positive history of breast cancer, a prior diagnosis of benign breast disease could plausibly make women more likely to present symptomatically between screens, and so this could also have contributed to some extent to the greater increase in the risk of interval cancer than screen-detected cancers in these women.

Although previously published studies have failed to demonstrate some of the significant associations observed here between key risk factors and mode of cancer detection, this could be due in part to a lack of precision in those studies, and in some cases, their findings were not inconsistent with ours. In addition, since none of the previously published studies presented findings restricted to never MHT users, it is difficult to ascertain the degree to which small associations of these risk factors with screening sensitivity might have been masked by the comparatively stronger, and well established, effect of MHT on screening sensitivity.

The main strength of our study is the large number of breast cancer outcomes during follow-up (about double that of the largest previous study), providing adequate power to reliably compare associations with prospectively diagnosed screen-detected and interval breast cancers. Other strengths include the prospective design of the study, which reduces the risk of recall bias, and the adjustment for the most relevant confounders. A further strength is the linkage to records from the NHSBSP. This allowed us to follow women only while they were attending for routine screening and, therefore, to control for confounding bias due to differential screening attendance.

Our study is not without limitations. As data on mode of detection of breast cancer were incomplete, we used the time between the start of a screening episode and diagnosis to discriminate between screen-detected and interval cancers which could have led to some degree of misclassification. However, the fact that the observed associations were similar regardless of whether a 6- or 9-month cut-off was used suggests that this is unlikely to have unduly influenced our findings. Another limitation was the lack of complete data on ER status. Although we were able to adjust risk factor associations for interval versus screen-detected cancers for grade, which is an important marker of tumour aggressiveness, we were unable to assess to what extent additional adjustment for ER status would have altered the main findings. Data on breast density was also lacking and we were unable to assess directly whether associations were mediated by density.

Overall, our findings suggest that women with low BMI, women with a first-degree family history of breast cancer and women with previous surgery for benign breast disease are likely to experience some reduction in mammographic screening sensitivity. These findings could help inform the development of more tailored breast screening programmes.

## Supplementary Material

Supplementary Appendix

## Figures and Tables

**Table 1:** Baseline characteristics of postmenopausal women who never used MHT and details of follow-up for interval and screen-detected breast cancers.

Baseline characteristic	
Number of women	517,555
Age, mean (SD)	57.3 (5.0)
Nulliparous; % (*n*)	11.6 (59,636)
Number of children in parous women; mean (SD)	2.5 (1.1)
Age at first birth in parous women; mean (SD)	24.1 (4.3)
Body mass index; mean (SD)	26.5 (4.9)
Alcohol drinks per week; mean (SD)	3.6 (5.0)
Current smoker; % (*n*)	19.3 (93,608)
First-degree family history with breast cancer; % (*n*)	10.8 (52,616)
Previous surgery for benign breast disease; % (*n*)	10.9 (56,135)
Follow-up	
Person-years (1000s)	5031
Number of screen-detected breast cancers	9421
Number of interval breast cancers	5166

Abbreviation: SD, standard deviation.

**Table 2:** Odds ratios (95% CIs) for interval versus screen-detected breast cancer in relation to tumour characteristics.

	Cases		Odds ratio (95% CI)	*p*-value
	Screen-detected	Interval		
Tumour size (mm)				
<20	5592	1744	1.00	<.001
20–49	1886	1900	3.18 (2.92, 3.46)	
50+	146	242	5.08 (4.10, 6.31)	
*Trend per 1 mm*			*1.05* (*1.05, 1.06*)	
Nodal involvement				
Negative	4855	1934	1.00	<.001
Positive	1429	1384	2.32 (2.11, 2.54)	
Stage				
1	3822	1288	1.00	<.001
2	1451	1295	2.58 (2.34, 2.85)	
3+	286	425	4.28 (3.63, 5.04)	
*Trend per unit of stage*			*2.25* (*2.10, 2.42*)	
Grade				
1	2246	499	1.00	<.001
2	4588	2118	2.12 (1.90, 2.37)	
3	1963	2051	4.58 (4.08, 5.15)	
*Trend per unit of grade*			*2.15* (*2.03, 2.27*)	
Histology				
Ductal	6997	3732	1.00	.001
Lobular	1044	667	1.23 (1.10, 1.37)	
Other	1380	767	1.02 (0.92, 1.12)	
Oestrogen receptor status				
Positive	2922	1477	1.00	<.001
Negative	386	470	2.27 (1.95, 2.64)	

*Note*: Characteristic (% missing): tumour size (21%); nodal involvement (34%); stage (41%); grade (8%); histological subtype (0%); oestrogen receptor status (64%). Odds ratios adjusted for age at the start of the breast screening episode, region and deprivation quintile. The *p*-values for tumour size, stage, and grade are based on trends.

Abbreviation: CI, confidence interval.

**Table 3:** Odds ratios (95% CIs) for interval versus screen-detected breast cancer in relation to established risk factors before and after adjustment for tumour grade.

	Cases			Adjusted		Additionally adjusted for tumour grade
	Screen-detected	Interval		Odds ratio (95% CI)		Odds ratio (95% CI)
Age at menarche (years)						
<12	3806	1991		1.00		1.00
12–13	2304	1247		1.02 (0.93, 1.12)		1.02 (0.93, 1.11)
14+	3135	1837		1.11 (1.02, 1.20)		1.09 (1.00, 1.19)
*Trend per year*				*1.03* (*1.00, 1.06*)		*1.03* (*1.00, 1.06*)
Parity						
Nulliparous	1239	696		1.00		1.00
1–2 children	5335	2923		1.10 (0.84, 1.43)		1.14 (0.87, 1.50)
3+ children	2804	1530		1.17 (0.89, 1.52)		1.20 (0.92, 1.58)
*Trend per child*				*1.06* (*0.98, 1.15*)		*1.06* (*0.97, 1.15*)
Age at first birth						
Nulliparous	1239	696		1.00		1.00
<25 years	5145	2795		1.08 (0.96, 1.21)		1.09 (0.96, 1.23)
25+ years	2811	1553		1.03 (0.91, 1.18)		1.04 (0.91, 1.19)
Body Mass Index (kg/m^2^)						
<25	3377	2175		1.00		1.00
25–30	3328	1732		0.83 (0.76, 0.90)		0.82 (0.76, 0.89)
30+	2152	969		0.72 (0.66, 0.80)		0.70 (0.63, 0.77)
*Trend per 5 kg/m^2^*				*0.85* (*0.81*, *0.89*)		*0.83* (*0.79, 0.88*)
Alcohol consumption (units/week)						
<3	5719	3039		1.00		1.00
3–7	1861	1091		1.05 (0.96, 1.14)		1.04 (0.95, 1.14)
8+	1763	984		1.00 (0.91, 1.10)		1.02 (0.93, 1.13)
*Trend per unit/week*				*1.00* (*1.00*, *1.00*)		*1.00* (*1.00*, *1.00*)
Smoking						
Never	4790	2680		1.00		1.00
Past	2298	1307		1.03 (0.94, 1.12)		1.06 (0.97, 1.16)
Current	1792	854		0.80 (0.72, 0.88)		0.83 (0.75, 0.91)
First-degree family history of breast cancer						
No	7473	3990		1.00		1.00
Yes	1355	865		1.20 (1.09, 1.32)		1.21 (1.10, 1.34)
Previous breast surgery						
No	8044	4167		1.00		1.00
Yes	1281	932		1.38 (1.25, 1.52)		1.38 (1.25, 1.52)

*Note*: Odds ratios adjusted for age at the start of the breast screening episode, region, deprivation quintile and the risk factors listed above.

Abbreviation: CI, confidence interval.

## Data Availability

Anonymised data used here can be accessed by application to the investigators and to the providers of follow-up data (e.g., NHS England) from any qualified investigator. The Million Women Study Data Access Policy can be viewed at [millionwomenstudy.org/data_ access]. Further information is available from the corresponding author upon request.

## What’s new?

In the United Kingdom, two thirds of breast cancers are detected at mammography. The remaining one third is found during the interval between screens. Here, using data from the Million Women Study, risk factor associations were compared for interval versus screen-detected breast cancer. Analyses show that body mass index had a smaller positive association with the risk of interval cancer than with the risk of screen-detected cancer. Both family history of breast cancer and previous surgery for benign breast disease had a greater positive assocition with the risk of interval cancer than with the risk of screen-detected cancer. The findings are consistent with the effects of these risk factors on mammographic screening sensitivity.
