# Supplementary material for: A comparative analysis of risk factor associations with interval and screen-detected breast cancers: A large UK prospective study
Source: Int J Cancer. Author manuscript; Available in PMC 2026 Oct 23. (PMC7619624; doi:10.1002/ijc.34968)
Supplement: Supplementary file 1 [file EMS217089-supplement-Supplementary_Appendix.pdf]

Supplementary Appendix

Supplementary Table 1. Odds ratios (95% CIs) for interval versus screen-detected breast cancer in relation to tumour characteristics. Interval and screen-detected breast cancers defined using time from start of breast screening episode to cancer registration using a cut-off of 6 months.

	Cases				
	Screen-detected	Interval	RR (95% CI)		p-value
**Tumour size (mm)**					
<20	4,950	2,386	1.00	(1.00, 1.00)	<0.001
20-49	1,647	2,139	2.65	(2.44, 2.88)	
50+	121	267	4.36	(3.48, 5.46)	
Trend per 1mm			*1.05*	*(1.04, 1.05)*	
**Node**					
Negative	4,429	2,360	1.00	(1.00, 1.00)	<0.001
Positive	1,228	1,585	2.29	(2.09, 2.51)	
**Stage**					
1	3,472	1,638	1.00	(1.00, 1.00)	<0.001
2	1,276	1,470	2.38	(2.16, 2.62)	
3+	231	480	4.28	(3.61, 5.08)	
Trend			*2.20*	*(2.05, 2.36)*	
**Grade**					
1	1,991	754	1.00	(1.00, 1.00)	<0.001
2	4,083	2,623	1.75	(1.59, 1.93)	
3	1,670	2,344	3.62	(3.25, 4.02)	
Trend			*1.93*	*(1.83, 2.03)*	
**Histology**					
Ductal	6,134	4,595	1.00	(1.00, 1.00)	0.011
Lobular	927	784	1.17	(1.05, 1.30)	
Other	1,177	970	1.07	(0.97, 1.17)	
**ER status**					
Positive	2,667	1,732	1.00	(1.00, 1.00)	<0.001
Negative	334	522	2.22	(1.90, 2.58)	

Characteristic (% missing): tumour size (21%); nodal involvement (34%); stage (41%); grade (8%); histological subtype (0%); oestrogen receptor status (64%). Odds ratios adjusted for age at the start of the breast screening episode, region and deprivation quintile. P-values for tumour size, stage and grade are based on trends. CI, confidence interval.

Supplementary Figure 1. Reciever operator characteristic curve for time (in months) from start of screening episode to cancer registration as a marker for interval versus screen-detected breast cancer

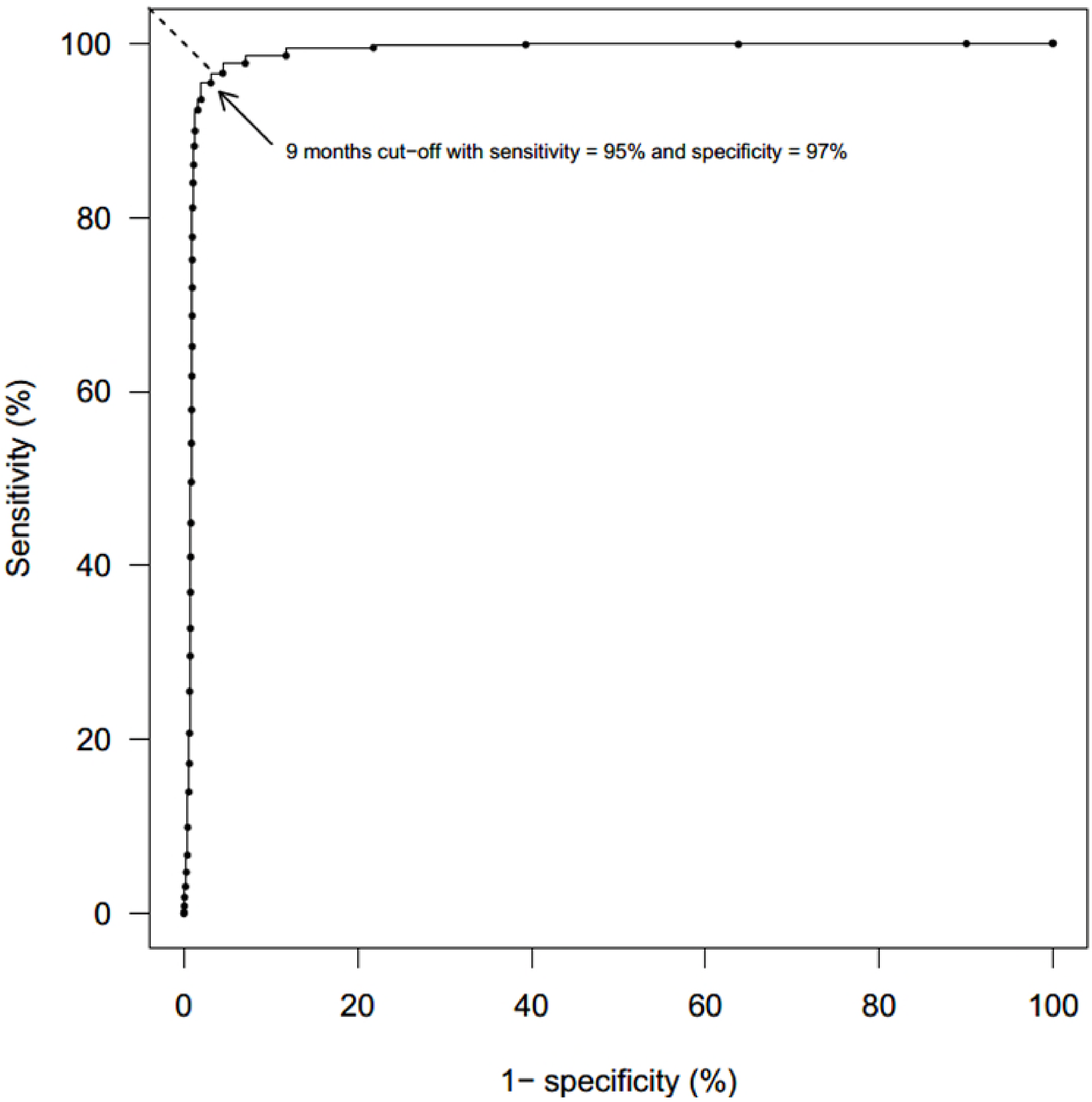

Supplementary Figure 2. Exemplars of follow-up for screen-detected breast cancers, interval breast cancers

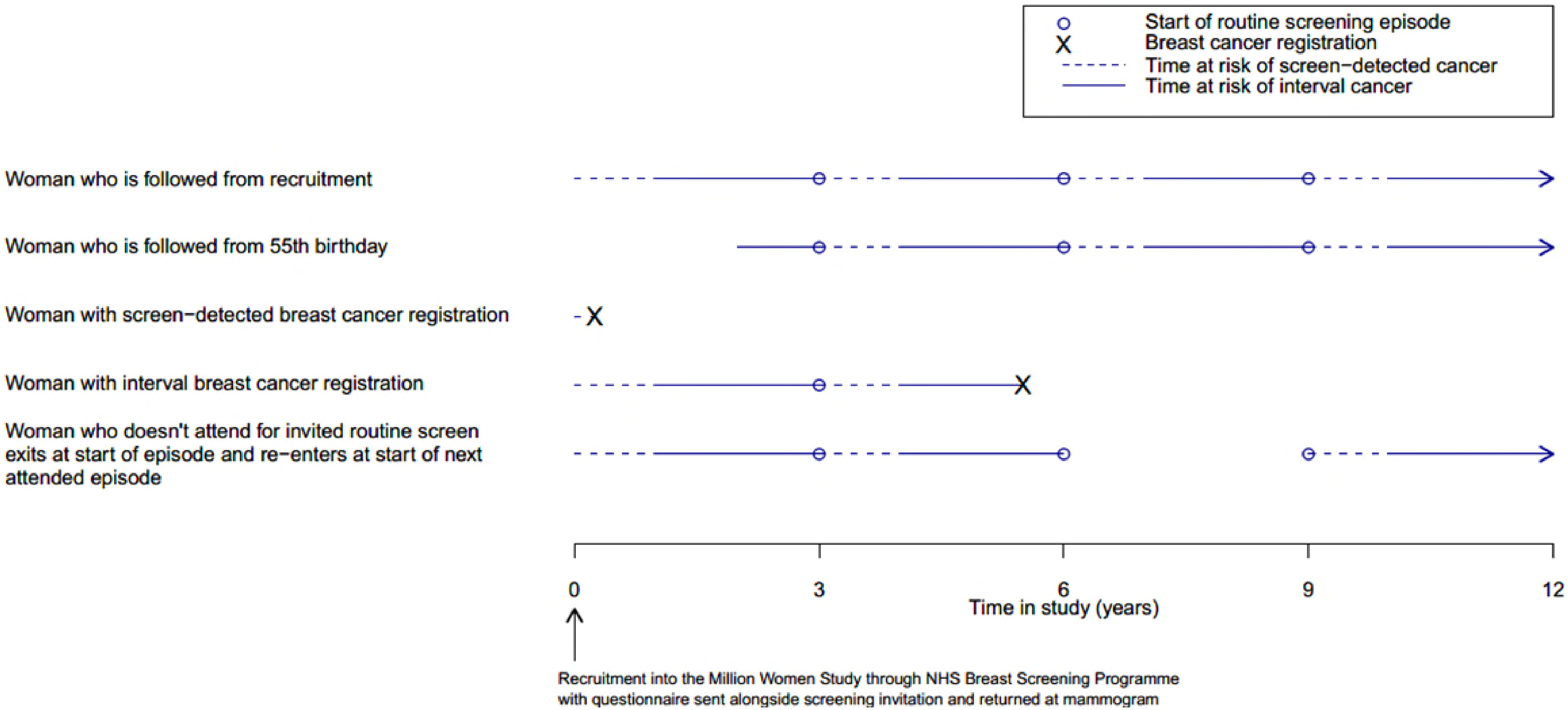

Supplementary Figure 3. Risk ratios (95%CIs) for interval and screen-detected breast cancer in relation to established risk factors. Interval and screen-detected breast cancers defined by time from start of breast screening episode to cancer registration using a cut-off of 6 months.

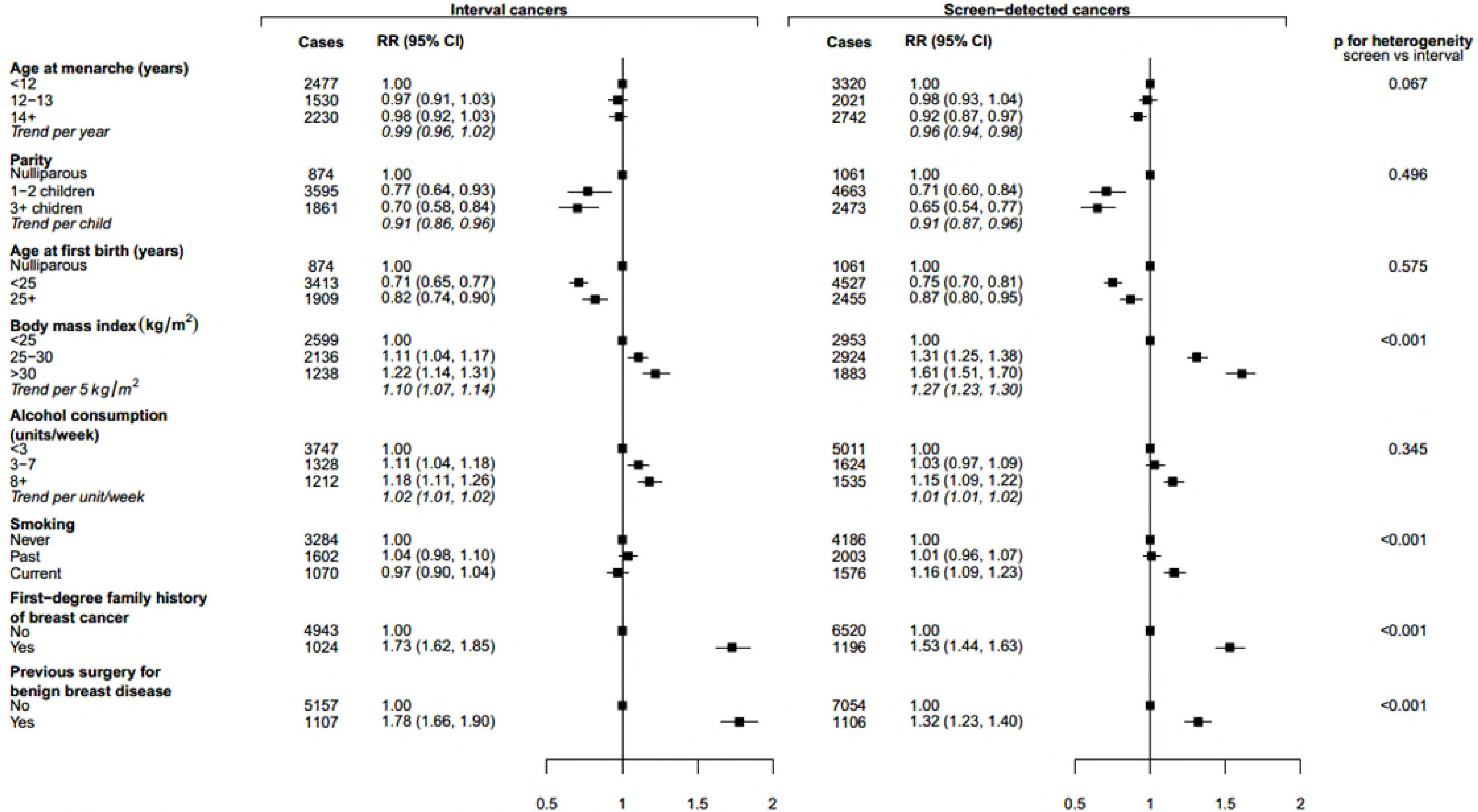

	Interval cancers: Cases	Interval cancers: RR (95% CI)	Screen-detected cancers: Cases	Screen-detected cancers: RR (95% CI)	p for heterogeneity screen vs interval
**Age at menarche (years)**					
<12	2477	1.00	3320	1.00	0.067
12–13	1530	0.97 (0.91, 1.03)	2021	0.98 (0.93, 1.04)	
14+	2230	0.98 (0.92, 1.03)	2742	0.92 (0.87, 0.97)	
Trend per year		*0.99 (0.96, 1.02)*		*0.96 (0.94, 0.98)*	
**Parity**					
Nulliparous	874	1.00	1061	1.00	0.496
1–2 children	3595	0.77 (0.64, 0.93)	4663	0.71 (0.60, 0.84)	
3+ children	1861	0.70 (0.58, 0.84)	2473	0.65 (0.54, 0.77)	
Trend per child		*0.91 (0.86, 0.96)*		*0.91 (0.87, 0.96)*	
**Age at first birth (years)**					
Nulliparous	874	1.00	1061	1.00	0.575
<25	3413	0.71 (0.65, 0.77)	4527	0.75 (0.70, 0.81)	
25+	1909	0.82 (0.74, 0.90)	2455	0.87 (0.80, 0.95)	
**Body mass index (kg/m^2)**					
<25	2599	1.00	2953	1.00	<0.001
25–30	2136	1.11 (1.04, 1.17)	2924	1.31 (1.25, 1.38)	
>30	1238	1.22 (1.14, 1.31)	1883	1.61 (1.51, 1.70)	
Trend per 5 kg/m^2		*1.10 (1.07, 1.14)*		*1.27 (1.23, 1.30)*	
**Alcohol consumption (units/week)**					
<3	3747	1.00	5011	1.00	0.345
3–7	1328	1.11 (1.04, 1.18)	1624	1.03 (0.97, 1.09)	
8+	1212	1.18 (1.11, 1.26)	1535	1.15 (1.09, 1.22)	
Trend per unit/week		*1.02 (1.01, 1.02)*		*1.01 (1.01, 1.02)*	
**Smoking**					
Never	3284	1.00	4186	1.00	<0.001
Past	1602	1.04 (0.98, 1.10)	2003	1.01 (0.96, 1.07)	
Current	1070	0.97 (0.90, 1.04)	1576	1.16 (1.09, 1.23)	
**First–degree family history of breast cancer**					
No	4943	1.00	6520	1.00	<0.001
Yes	1024	1.73 (1.62, 1.85)	1196	1.53 (1.44, 1.63)	
**Previous surgery for benign breast disease**					
No	5157	1.00	7054	1.00	<0.001
Yes	1107	1.78 (1.66, 1.90)	1106	1.32 (1.23, 1.40)	

Relative risks stratified by year of birth, year of recruitment; adjusted for region, deprivation quintile and risk factors listed above. P–values for heterogeneity are based on trends for age at menarche, parity, body mass index, and alcohol consumption. RR, relative risk; CI, confidence interval.
